# Effects of Imunit Insecticide on Biological Characteristics and Life Table Parameters of *Spodoptera cilium* (Lepidoptera: Noctuidae)

**DOI:** 10.3390/insects12121138

**Published:** 2021-12-20

**Authors:** Marzieh Hatami, Masumeh Ziaee, Ali Asghar Seraj, Mehdi Mehrabi-Koushki, Jacek Francikowski

**Affiliations:** 1 Department of Plant Protection, Faculty of Agriculture, Shahid Chamran University of Ahvaz, Ahvaz 61357-43311, Iran; m-hatami@stu.scu.ac.ir (M.H.); seraj.a@scu.ac.ir (A.A.S.); mhdmhrb@scu.ac.ir (M.M.-K.); 2 Laboratory of Insect Physiology and Ethology, Institute of Biology, Biotechnology and Environmental Protection, Faculty of Natural Sciences, University of Silesia in Katowice, 9 Bankowa Street, 40-007 Katowice, Poland

**Keywords:** grass, insecticide, life table, *Spodoptera cilium*

## Abstract

**Simple Summary:**

*Spodoptera cilium* Guenee (Lepidoptera: Noctuidae) is one of the grass pests in some parts of the world, including the southern regions of Iran. The larvae of *S.* *cilium* feed on grasses and heavy infestations can severely destroy lawn grasses. In the present study, we monitored the effects of Imunit on some biological and demographic parameters of the offspring generation. Our results indicate that Imunit reduced the survival rate and fecundity of *S. cilium* and could be used in the management programs of this pest.

**Abstract:**

Imunit is a mixture of alpha-cypermethrin + teflubenzuron, and has been launched for controlling caterpillars. In this study, the effects of Imunit at LC_50_ and LC_30_ were investigated on parental and offspring generation of *S. cilium*, according to age-stage, two-sex life table. The experiments were conducted by leaf dipping method at 25 °C and 60 ± 5% relative humidity, under a cycle of 16 h fluorescent light and 8 h darkness. LC_30_ and LC_50_ concentrations of Imunit increased the immature developmental time of *S. cilium* in the offspring generation, while the LC_50_ of Imunit significantly reduced the developmental time of adults. The adult pre-oviposition period and total pre-oviposition period considerably increased when offspring were treated with LC_50_ of Imunit. In offspring of *S. cilium* exposed to LC_50_ and LC_30_ concentrations of Imunit, the gross reproductive rate (*GRR*), net reproduction rate (*R*_0_), the intrinsic rate of population increase (*r*), and the finite rate of population increase (*λ*) significantly reduced compared to the control. This study showed that the application of Imunit at LC_50_ could suppress the *S. cilium* population and can be used in the integrated management program of this pest.

## 1. Introduction

The genus *Spodoptera* moths migrate in clusters and feed on numerous plants. Due to the wide range of hosting and global distribution, *Spodoptera* species are important pests of crops. Adults of *Spodoptera* are active at night and classify as a nocturnal pest, but their larvae are active and feeding on plants during the day [1]. The grass-lawn armyworm *Spodoptera cilium* Guerine (Lepidoptera: Noctuidae) is considered a major pest of Graminaceous plants [2], and classified as the main limitation of rice yield in African countries [3].

The indiscriminate use of insecticides, including chemical and bio-insecticides, especially those with a wide range, has led to ecological imbalances, environmental hazards, severe disruptions to natural and agricultural ecosystems. Additionally, increasing insecticide pressure leads to pest resistance to many typical insecticides, resulting in pests’ re-emergence [4,5]. Sub-lethal concentrations affects the biological characteristics, physiology, behavior, and parameters of the life table of insects. For example, sub-lethal concentrations affect the population growth of insects and their life table parameters [6]. Several biological effects of sub-lethal concentrations of insecticides have been reported in the literature [7,8,9]. Sub-lethal effects of the pesticides including lufenuron, methoxyfenozide, spinosad, endosulfan, novaluron, and tebufenozide resulted in the reduction in pupal weight, adult developmental period time, and fertility of *Anticarsia gemmatalis* Hübner (Lepidoptera: Noctuidae) [7]. The demographic parameters of *Plutella xylostella* L. (Lepidoptera: Plutellidae), in several generations, was affected by the LC_25_ concentration of the insecticide spinosad [8]. The insecticide hexaflumuron reduced the total number of eggs laid by each female, oviposition period, and adult emergence time of *P. xylostella* [10]. Reproductive life tables for LC_10_, and LC_25_ concentrations of the insecticide chlorantraniliprole on diamondback moth illustrated that the developmental period of the larvae and pupae increase after using this pesticide [11]. The sub-lethal effects of spinosad can affect the population dynamics of *Spodoptera exigua* Hübner (Lepidoptera: Noctuidae) by reducing the survival rate, reproduction, and development time of this species [9]. Effects of cyantraniliprole at LC_30_ reduced the pupal weight and fertility of *Helicoverpa assulta* Guenée (Lepidoptere: Noctuidae). However, this insecticide did not significantly affect the development period of pupae and adult emergence [12]. The effectiveness of five insecticides was evaluated to control *S. cilium* under laboratory and field conditions in the local area of lawns. *Spodoptera* *cilium* created brown patches and bare spots in the lawn. However, among the tested insecticides, Imunit was the most active against grass-lawn armyworms and reduced the damage percentage (28%) of this pest [13]. 

Imunit 150 SC is a mixture of alpha-cypermethrin (7.5%) and teflubenzuron (7.5%). According to data from the Insecticide Resistance Action Committee (IRAC) classification, alpha-cypermethrin belongs to the sodium channel modulating neurotoxins (group 3A) and the subtype of pyrethroids. Teflubenzuron is in the IRAC classification member of group 15, which be included in the class of insect growth regulators and inhibitors of chitin biosynthesis and the benzene-urea subgroup [14].

The purpose of the current study was to evaluate the effect of LC_50_ and LC_30_ concentrations of Imunit on *S. cilium* through investigating the biological characteristics and life table parameters of the pest. 

## 2. Materials and Methods

### 2.1. Rearing of Spodoptera Cilium

The larvae of *S. cilium* were collected from infested green-lawn spaces in Gachsaran, Iran, in July 2019. Collected larvae were fed on grass leaves until the emergence of adult insects and identified based on morphological characters. A mixture of 50% Bermudagrass (*Cynodon dactylon* (L.) Pers.), and 50% Sport grass (including 80% *Lolium perenne* L., 10% *Festuca rubra* cv. Reverent, 10% *Poa pratensis* L.) seeds were planted in the greenspace in Dogonbadan Municipality, Gachsaran, and used for rearing the grass-lawn armyworms. Collected larvae were introduced to cylindrical plastic containers (15 cm in diameter and 15 cm in height) and grass leaves were provided to the larvae daily. The lids of the containers were enclosed with piece of muslin for ventilation. Each container was set down in the growth chamber regulated at 25 ± 1 °C, 60 ± 5% relative humidity (RH), and a cycle of 16 h fluorescent light and 8 h dark. Adult moths were fed with a 20% honey solution impregnated onto cotton wool. Field collected insects were reared for three generations to acclimate the insects to laboratory conditions. Third-instars from the fourth generation were used in the experiments.

### 2.2. Insecticide Tested

Imunit (150 SC) is a mixture of alpha-cypermethrin (7.5%) and teflubenzuron (7.5%), and was supplied by BASF, Ludwigshafen, Germany.

### 2.3. Dose-Mortality Response Bioassay

Bioassays were conducted using the leaf dip method. Grass leaves (two grams/dish) were dipped in different concentrations (25, 50, 90, 150, and 180 ppm) of the Imunit insecticide solution for 10 s. Distilled water was served as a control. The treated leaves were air-dried for one hour at room temperature and placed in plastic Petri dishes (12 cm in diameter) with a hole (2 cm in diameter) capped with a muslin cloth. In each Petri dish, 25 third-instar larvae were released, and the experiment was replicated three times. Percentage larval mortality was counted 48 h after treatment using a Nikon SMZ800 stereomicroscope (Japan). All experiments were performed in the growth chamber set at 25 ± 1 °C, 60 ± 5% RH, and 16:8 h (L:D) photoperiod. 

### 2.4. Imunit Effects on Parental Generation

Grass leaves (two grams/dish) were dipped in LC_30_ (18.2 ppm) and LC_50_ (27.7 ppm) concentrations of Imunit insecticide solution for 10 s. Distilled water served as a control. The treated leaves were air-dried for one hour at room temperature and placed in plastic Petri dishes (12 cm in diameter) with a hole (2 cm in diameter) capped with a muslin cloth. Then, 60 third-instar larvae were released separately into each Petri dish and kept under experimental conditions for 48 h. After 48 h, the surviving larvae were separately transferred to untreated leaves in plastic Petri dishes. The Petri dishes were checked daily until pupation, and developmental time was recorded for all immature stages sing a Nikon SMZ800 stereomicroscope (Japan). After the moth imago emergence, females were coupled with males obtained from the selfsame treatment in separate plastic containers (11 cm length × 9 cm width × 4 cm height) containing 20% honey solution as food. The adults were transferred to new containers every day, and the total number of eggs laid was counted daily. This procedure was continued until all the adults died. All experiments were performed in the growth chamber set at 25 ± 1 °C, 60 ± 5% RH, and 16:8 h (L:D) photoperiod. 

### 2.5. Imunit Effects on Offspring Generation 

To assess the effects of Imunit to the offspring generation, 100 eggs produced from parents were transmitted separately to the test Petri dishes containing 2 g grass leaves. Fresh leaves (two grams/dish) were added daily to the dishes, and the immature development time was recorded every day. As mentioned above, when adults emerged, female moths were coupled with males, and if the male died before the female, a new male was transferred to the container. 

### 2.6. Data Analysis

Data were subjected to Probit analysis (Finney 1971) to estimate LC_30_ and LC_50_ concentrations of Imunit using SAS 6.12 software (SAS Institute 1997). The development time of the third- to fifth-instar larvae, pupae, adult, and oviposition period of the parental generation and also, the development time of the offspring generation including duration of eggs, first- to fifth-instar larvae, pupae, pre-adult, adult, oviposition period, adult pre-ovipositional period (APOP), and total pre-ovipositional period (TPOP) were analyzed using the age–stage, two-sex life table [15,16] using TWOSEX MS Chart software [17]. From the output of this software we extracted the following parameters: Age-specific survival rate (*l_x_*), Age-specific fecundity of total population (*m_x_*), Gross reproductive rate (*GRR*), net rate of reproduction (*R*_0_), intrinsic rate of population growth (*r*), finite rate of population growth (*λ*), and the average period length of one generation (*T*). The intrinsic rate of population growth was obtained from the Euler–Lotka formula, with age indexed from zero, according to Goodman [18]. Age–stage-specific life expectancy (*exj*) (*x*: insect age (days) and *j*: insect growth stage (period)) was also calculated. The mean and standard error of life table indices were estimated using the Bootstrap method [19]. The paired Bootstrap test based on confidence limits was used to compare the means. 

## 3. Results

### 3.1. Estimation of Concentrations

The LC_30_ and LC_50_ concentrations of Imunit on third instar larvae were 18.2, and 27.7 ppm, respectively (Table 1). 

### 3.2. Imunit Effects on Parental Generation 

The results of parental generation (F0) treatment indicated that there were no significant differences in developmental time of third- and fifth-instar larvae of *S. cilium* after being treated with LC_30_ and LC_50_ of Imunit in comparison with the control group. In contrast, the development time of pupae was significantly longer in the treatment group compared to the control group. However, the time of adult emergence and oviposition period were significantly increased in the LC_30_ and LC_50_-treated group of Imunit compared to the control (Table 2). 

### 3.3. Imunit Effects on Offspring Generation 

For offspring generation (F1), the pre-adult development time was significantly prolonged on the LC_30_ (34.56 d) and LC_50_ treatments (37.67 d) of Imunit in comparison to the control (29.63 d). However, the adult and oviposition periods were significantly shorter on the LC_30-_ and LC_50_-treated groups of Imunit. This insecticide significantly extended the APOP, TPOP, and female longevity at LC_30_ and LC_50_ concentrations compared with the control. However, the male longevity of *S. cilium* was not affected by the LC_30_ concentration of Imunit compared to the control group (Table 3). 

The results demonstrated that the LC_30_ and LC_50_ of Imunit significantly reduced gross reproductive rate (*GRR*), net reproductive rate (*R_0_*), intrinsic rate of population growth (*r*), and finite rate of population increase (*λ*) compared to the control. The mean generation time of offspring generation (F1) showed a significant increase in the LC_30_- (38.78 d) and LC_50_-treated (42.0 d) group of Imunit compared to the control (33.87 d) (Table 4).

**Figure 1:**
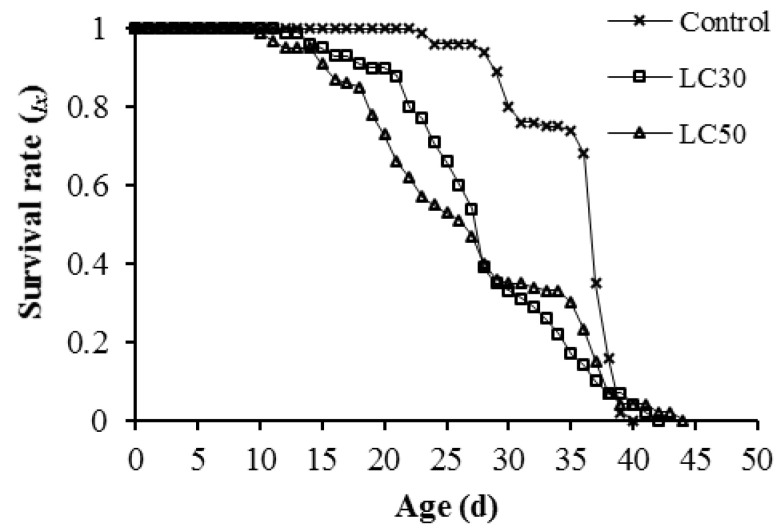
Age-specific survival rate (*l_x_*) of *Spodoptera cilium* of offspring generation (F1) exposed to LC_30_ and LC_50_ concentrations of Imunit.

**Figure 2:**
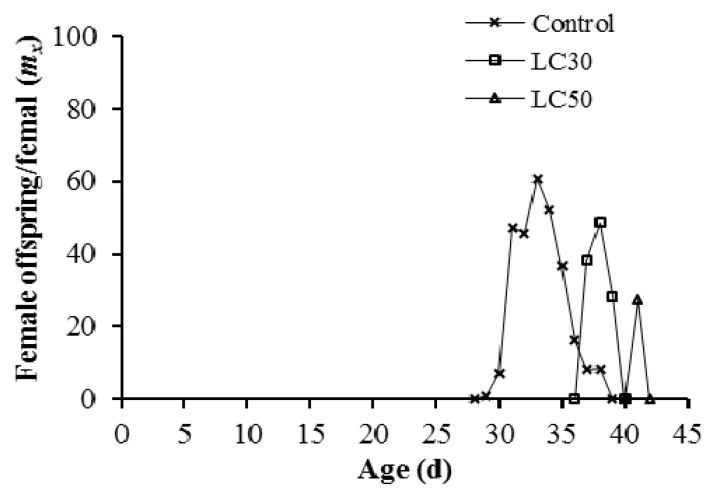
Age-specific fecundity of total population (*m_x_*) produced by *Spodoptera cilium* of offspring generation (F1) exposed to LC_30_ and LC_50_ concentrations of Imunit.

**Figure 3:**
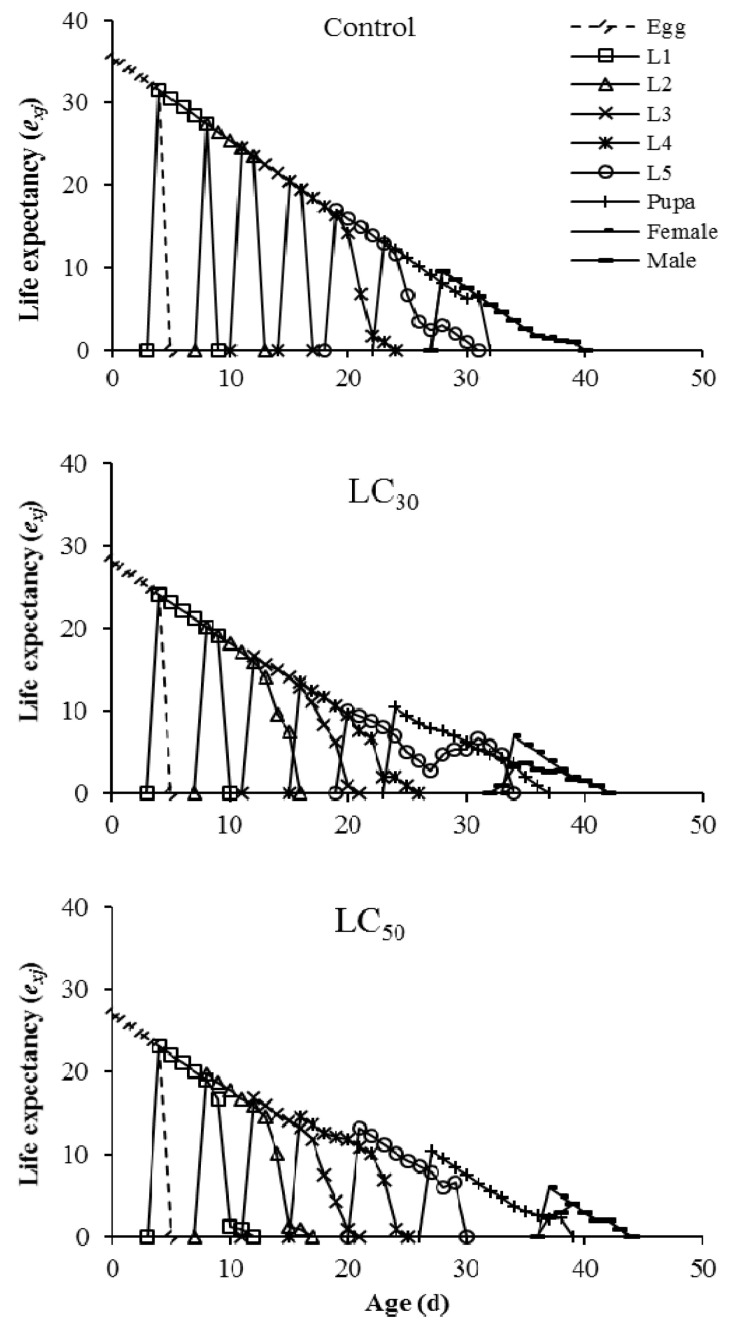
Age–stage specific life expectancy (*e_xj_*) of *Spodoptera cilium* offspring generation (F1) exposed to LC_30_ and LC_50_ concentrations of Imunit.

The age-specific survival rate (*l_x_*) indicated a slight decrease within the first 18 and 21 d in LC_30_ and LC_50_ treatments, respectively (Figure 1). The fecundity level in the LC_30_ and LC_50_ treatments was lower than in the control. The highest mean number of hatched eggs produced by each individual was reported on day 33 (60.72 eggs) in the control group (Figure 2). The number of days that an individual of age *x* and stage *j* is expected to survive is shown by the age-specific life expectancy curve (*exj*). In offspring generation (F1), the *exj* in the control treatment was more than theLC_30_ and LC_50_-treated groups (Figure 3).

## 4. Discussion

Limiting the number and concentration of pesticides used is one of the important goals of integrated pest management (IPM), reducing the risks to human health, the environment, and beneficial insects [20]. It is necessary to find an insecticide that can sufficiently reduce the population and damage of grass-lawn armyworm *S. cilium*. In our previous study, Imunit had the highest insecticidal activity on larvae of *S. cilium* compared to other tested insecticides, including indoxacarb, fenvalerate, diflubenzuron, and a commercial formulation of the bacterium *Bacillus thuringiensis* [13]. Accordingly, Imunit was selected from the typical insecticides used against *S. cilium* to conduct an investigation into the sub-lethal effects on biological characteristics and life table parameters of this pest. 

Our results showed that Imunit used in LC_30_ concentration significantly extended the developmental time of *S. cilium* pre-adult stages but decreased the adults’ duration in the offspring generation. One of the active ingredients of Imunit is teflubenzuron, a chitin biosynthesis inhibitor in the benzoylurea class that interferes with the molting of insects [21]. The inhibitory activity of teflubenzuron on the formation of chitin has been reported in *Spodoptera littoralis* Boisduval [22]. Very little has been known about the sublethal effects of teflubenzuron on the population and demographic parameters of different species of the genus *Spodoptera* (Lepidoptera: Noctuidae). Methoxyfenozide is an ecdysone receptor agonist (diacylhydrazine class), and at LC_25_ significantly increased larval and pupal growth time of *S. exigua*. The increase of larval development time may be due to the induction of additional molting in larvae, which arises from the use of ecdysone agonists [23]. Hexaflumuron (benzoylurea class) at LC_10_ and LC_25_ concentrations prolonged the development duration of pre-adult stages, rather than shortening the adults’ longevity. Moreover, hexaflumuron at sublethal concentrations decreased the fecundity of *P. xylostella* [24]. However, diflubenzuron, another insecticide of the benzoylurea class, did not affect the growth and reproduction of *Platynota idaeusalis* Walker (Lepidoptera: Tortricidae), but it increased the mortality of male pupae. Diflubenzuron was reported as the weakest insecticide in its class [25]. Lufenuron, another benzoylurea insecticide, at LC_50_ decreased the size and weight of *P. xylostella* pupae, but the larval and pupal growth period was not affected. Moreover, lufenuron significantly reduced the adult emergence [26]. Teflubenzuron inhibited larval molting, larval-pupal ecdysis and adult emergence of *Leptinotarsa decemlineata* (Say). Teflubenzuron was noted to have antifeedant activity and reduced larval foliage-feeding, which leads to a reduction in the consumption rate of the larvae and their weakening. Moreover, teflubenzuron ingestion delayed the development of *L. decemlineata* larvae resulted in prolongation of larval period [27]. The larvae of *Agrotis ipsilon* (Hufnagel) (Lepidoptera: Noctuidae) exposed to the insecticide, cyantraniliprole, expend more energy on detoxification than on growth, thus increasing their developmental duration compared to the control [28].

In our study, the oviposition period and fecundity of both parent and offspring generations were significantly decreased at LC_30_ and LC_50_ concentrations of Imunit. Azadirachtin reduced fecundity in African armyworm, *Spodoptera exempta* Walker at doses of 0.01 and 0.1 µg of the active ingredient per larva as a topical application [29]. Similar effect was observed in *S. littoralis* when this insecticide was added to the diet of larvae at 0.001 ppm concentration [30]. Azadirachtin and methoxyfenozide (ecdysone agonist) at 100 mg (a.i) per liter influenced the population dynamics of *S. littoralis* and significantly reduced adult longevity, fecundity, and fertility [31]. Teflubenzuron, like other chitin synthesis inhibitors, adversely affects the fecundity of moths [25,26].

The life table parameters *GRR*, *R*_0_, *r*, and *λ* decreased significantly in the progeny generation of *S. cilium* compared to the control group. Similar outcomes were reported by [9], when spinosad was applied in food of *S. exigua* at a sublethal concentration of 0.30 mg (a.i) per kg diet weight. This concentration significantly reduced the *R*_0_ and *r* values in comparison to the control group. Similarly, demographic growth parameters (*R*_0_, *r*, and *λ*) of *P. xylostella* offspring generation were significantly reduced when treated with LC_25_ of chlorantraniliprole in comparison with the control [32]. Our results also illustrated that Imunit, even at sublethal concentration, had an adverse influence on other parameters such as *l_x_*, *m_x_*, and *e_xj_*, indicating Imunit inhibited the survival rate and life expectancy of *S. cilium.*

Another active ingredient in this insecticide is alpha-cypermethrin. Cypermethrin at LC_10_, LC_30_, and LC_50_ concentrations significantly decreased adult longevity, fecundity, fertility, as well as adult emergence of *Helicoverpa armigera* Hübner (Lepidoptera: Noctuidae). Furthermore, demographic growth parameters of the chickpea pod borer, *H. armigera*, including *R*_0_, *r*, and *λ* are negatively affected by cypermethrin [33]. Beta-cypermethrin at LC_15_, LC_30_, and LC_50_ values significantly prolonged the developmental duration of *P. xylostella* larvae than the control group. The oviposition period of offspring generation was shortened, and a significant fecundity reduction was detected in LC_10_, and LC_30_, treated groups. Moreover, mean values of *r*, *λ*, *GRR*, and *R*_0_ were significantly lower in all the treated groups than in the untreated control group [34]. These results are in accordance with our findings. Imunit negatively affected population growth and demographic parameters of *S. cilium.* Quan et al. [35] reported that beta-cypermethrin at LC_10_ significantly reduced fertility and adult longevity of *Carposina sasakii* Matsumura (Lepidoptera: Carposinidae). The age-specific survival rate (*l_x_*) of the adults was also adversely affected by the LC_10_ concentration of beta-cypermethrin, but this insecticide increased the duration of the oviposition period and stimulated fecundity [35]. 

## 5. Conclusions

The present study suggests that both active ingredients in Imunit (alpha-cypermethrin + teflubenzuron) can effectively reduce the grass-lawn armyworm, *S. cilium* population. Imunit suppress the population growth of *S. cilium.* Imunit, at low concentrations, can be considered in integration with other low-risks methods for controlling *S. cilium* in green spaces.

## Figures and Tables

**Table 1:** Toxicity of Imunit on third-instar larvae of *Spodoptera cilium*.

Insecticide	Number of Larvae Tested	df	LC_50_ (ppm)	LC_30_ (ppm)	Chi Square	*p* Value
Imunit	375	3	27.7	18.2	3.60	0.40

**Table 2:** Effects of Imunit on developmental time, and fecundity of female adult (Mean ± SE) of *Spodoptera cilium* parental generation (F0).

Life Stage	Treatments		
	Control	LC_30_	LC_50_
Third-instar larvae (d)	4.00 ± 0.041 a	4.10 ± 0.03 a	4.08 ± 0.3 a
Fourth-instar larvae (d)	3.93 ± 0.042 b	4.06 ± 0.03 a	4.03 ± 0.02 a
Fifth-instar larvae (d)	4.15 ± 0.08 a	4.07 ± 0.05 a	4.05 ± 0.05 a
Pupae (d)	5.23 ± 0.06 c	6.48 ± 0.17 b	7.17 ± 0.19 a
Adult (d)	8.28 ± 0.17 a	7.52 ± 0.31 a	5.61 ± 0.43 b
Oviposition (d)	4.83 ± 0.23 a	3.93 ± 0.25 b	3.40 ± 0.45 b
Fecundity (eggs/female)	837.4 ± 22.54 a	514.3 ± 43.89 b	434.3 ± 24.95 c

Standard errors were estimated by using the bootstrap technique with 100,000 resampling. Means followed by the same letter within a row are not significantly different using the paired bootstrap test based on the confidence interval of difference (*p* < 0.05).

**Table 3:** Effects of Imunit on developmental time (Mean ± SE) of *Spodoptera cilium* offspring generation (F1).

Life Stage	Treatments		
	Control	LC_30_	LC_50_
Egg (d)	4.03 ± 0.04 c	4.20 ± 0.04 b	4.78 ± 0.04 a
First-instar larvae (d)	4.02 ± 0.01 c	4.28 ± 0.04 b	4.44 ± 0.05 a
Second-instar larvae (d)	4.09 ± 0.03 b	4.22 ± 0.04 b	4.62 ± 0.05 a
Third-instar larvae (d)	4.03 ± 0.03 a	4.08 ± 0.03 a	4.12 ± 0.05 a
Fourth-instar larvae (d)	4.07 ± 0.04 c	4.42 ± 0.06 b	4.96 ± 0.26 a
Fifth-instar larvae (d)	4.16 ± 0.04 b	4.55 ± 0.27 b	5.23 ± 0.10 a
Pupae (d)	5.33 ± 0.05 b	9.88 ± 0.12 a	9.50 ± 0.34 a
Pre-adult (d)	29.63 ± 0.09 c	34.56 ± 0.18 b	37.67 ± 0.33 a
Adult (d)	7.91 ± 0.08 a	3.81 ± 0.53 b	3.83 ± 0.91 b
Oviposition (d)	5.61 ± 0.17 a	2.00 ± 0.00 b	1.00 ± 0.00 c
APOP ^1^ (d)	1.14 ± 0.07 c	2.67 ± 0.33 b	3.33 ± 0.33 a
TPOP ^2^ (d)	30.61 ± 0.16 c	37.33 ± 0.33 b	41.00 ± 0.00 a
Fecundity (eggs/female)	728.21 ± 85.70 a	306.33 ± 82.28 b	195.0 ±55.0 c
Adult longevity (male) (d)	37.52 ± 0.17 b	37.77 ± 0.68 b	40.75 ± 1.38 a
Adult longevity (female) (d)	37.57 ± 0.21 c	41.00 ± 0.58 b	43.00 ± 0.57 a
Total longevity (all individuals) (d)	35.43 ± 0.407 a	28.18 ± 0.690 b	27.09 ± 0.88 b

Standard errors were estimated by using the bootstrap technique with 100,000 resampling. Means followed by the same letter within a row are not significantly different using the paired bootstrap test based on the confidence interval of difference (*p* < 0.05). ^1^ Adult pre-oviposition period, time between adult emergence and first oviposition. ^2^ Total pre-oviposition period, time from birth to first reproduction in female.

**Table 4:** Effects of Imunit on the population parameters (Mean ± SE) of *Spodoptera cilium* offspring generation (F1).

Parameters	Treatments		
	Control	LC_30_	LC_50_
*GRR* (offspring/female)	282.9 ± 52.75 a	114.9 ± 55.65 b	97.5 ± 51.41 b
*R*_0_ (offspring/female)	203.9 ± 40.15 a	9.190 ± 5.32 b	3.900 ± 2.58 b
*r* (d^−1^)	0.160 ± 0.01 a	0.050 ± 0.02 b	0.032 ± 0.01 c
λ (d^−1^)	1.170 ± 0.01 a	1.050 ± 0.02 b	1.030 ± 0.01 b
*T* (d)	33.87 ± 0.27 c	38.78 ± 0.26 b	42.00 ± 0.02 a

Standard errors were estimated by using the bootstrap technique with 100,000 resampling. Means followed by the same letter within a row are not significantly different using the paired bootstrap test based on the confidence interval of difference (*p* < 0.05).

## Data Availability

The data presented in this study are available on request from the M.Z.
